# Supplementary material for: Economic complexity unfolded: Interpretable model for the productive structure of economies
Source: PLoS One. 2018 Aug 7;13(8):e0200822. doi: 10.1371/journal.pone.0200822 (PMC6080758; doi:10.1371/journal.pone.0200822)
Supplement: S1 Supporting Information — Additional description for the results presented in the manuscript: Data DescriptionIndian Buffet Process and extensionsSparse Three-parameter Restricted IBPInference and SettingsRobustness Results (PDF) [file pone.0200822.s001.pdf]

S1 Supporting Information – Economic Complexity Unfolded: Interpretable Model for the Productive Structure of Economies

A Data Description

A.1 Trade Data

To empirically analyse the performance of our model we use the UN COMTRADE Standard International Trade Classification (SITC) rev.2 dataset, which disaggregates products to the four digit level, provided by the team of the Observatory of Economic Complexity. We focus our analysis on the year 2010. In order to clean unreliable or inadequately classified data, we restrict the dataset to the same countries that were used in the Atlas of Economic Complexity [5]. This leaves us with data on 126 countries and 744 products.

For the robustness check, we use the Harmonized System (HS) rev. 1992 classification disaggregated to six digit level (4890 products). The original data was collected by UN COMTRADE, and was further cleaned by the team of the Observatory of Economic Complexity (the HS data were also cleaned by the BACI team). They are available at `http://atlas.media.mit.edu/en/resources/data/`.

A.2 Country-Product Network

Consider the graph $\mathcal{G} = (\mathcal{C}, \mathcal{P}, \mathcal{E})$ in which the vertices are partitioned into two disjoint sets: the set of countries $\mathcal{C}$ of cardinality C, and the set of products $\mathcal{P}$ exported by the countries, of cardinality P. An edge (c, p) between a country $c \in [C]$ and a product $p \in [P]$ is present in the set of edges $\mathcal{E}$ if the country has a revealed comparative advantage in the export of that product:

$$M_{cp} = \begin{cases} 1, & \text{if } \mathrm{RCA}_{cp} \geq 1 \\ 0, & \text{otherwise} \end{cases} . \tag{1}$$

In (1) RCA_{cp} denotes Balassa's RCA index defined as:

$$\mathrm{RCA}_{cp} = \frac{E_{cp} / \sum_p E_{cp}}{\sum_c E_{cp} / \sum_{c,p} E_{cp}}, \tag{2}$$

where E_{cp} is the export of product p by country c. $\mathrm{RCA}_{cp} > 1$ indicates that country c's share of product p is larger than the product's share of the entire world market, thus "revealing" a comparative advantage of the country in the corresponding product.

The diversity (diversification) d_c of a country c is defined as the number of products in which it has comparative advantage, i.e. its degree in the network:

$$d_c = \sum_p M_{cp}, \tag{3}$$

Similarly, the ubiquity u_p of a product is the number of countries that produce that product:

$$u_p = \sum_c M_{cp}. \tag{4}$$

B Indian Buffet Process and extensions

B.1 Indian Buffet Process

The Indian buffet process (IBP) is a stochastic process defining a probability distribution over equivalence classes of sparse binary matrices with a finite number of rows and unbounded number of columns [4]. Although the number of columns is potentially infinite, only a finite number of those will contain non-zero entries due to the finite nature of the

observed data. Another important property of IBP-generated matrices is that they are exchangeable both in rows and columns, i.e., the order of the rows and columns are irrelevant. The IBP can be derived taking the limit as $K \to \infty$ of a finite binary matrix $\mathbf{Z} \in \{0,1\}^{C \times K}$, where C is the number of observations, and K is the number of latent features. Each element Z_{ck} is distributed according to:

$$\begin{aligned} \pi_k &\sim \text{Beta}(\alpha/K, 1), \\ Z_{ck} &\sim \text{Bernoulli}(\pi_k), \end{aligned} \tag{5}$$

where π_k is the probability of observing a non-zero value in column k and $\mathbf{Z}_{c\bullet}$ is the c-th row representing sample c. We say that a feature k is active for sample c if $Z_{ck} = 1$. When $K \to \infty$, the above finite model tends to the IBP, denoted by:

$$\mathbf{Z} \sim \text{IBP}(\alpha), \tag{6}$$

where α is the mass parameter controlling the a priori activation probability of new features. Alternatively, the IBP can also be constructed based on its underlying De Finetti's representation, i.e., as a mixture of Bernoulli processes directed by a beta process[1]

$$\mu \sim \text{BP}(1, \alpha, H), \tag{7}$$

$$\mathbf{Z}_{c\bullet} \sim \text{BeP}(\mu), \tag{8}$$

where μ is the directing measure, and H is the probability base measure for the beta process [9].

In the IBP, the number of active features per row is distributed according to Poisson(α) and the total number of active features K^+, i.e., number of columns with non-zero entries, is distributed as Poisson$\big(\alpha \sum_{i=1}^{C} \left(\frac{1}{i}\right)\big)$. The single scalar parameter α has thus an effect on both sparsity density and sparsity structure of the latent matrix $\mathbf{Z}$. Such assumption might not always be appropriate, specially in data exploration applications where we expect a high number of latent features and a different sparsity degree per row in the latent matrix.

B.2 Three-Parameter Indian Buffet Process

In the three-parameter IBP, the stick weights follow a more flexible distribution that cover power-law behaviors [8]. This can be achieved by replacing the beta process directing measure in the IBP by a stable-beta process. As its name indicates, this process can be fully specified by three parameters: α is the same mass parameter from the IBP that controls the *a priori* expected total number of non-zero entries in matrix $\mathbf{Z}$. Additionally, the stability exponent $\sigma \in [0, 1)$ controls the power-law behavior of the model, and $\delta > -\sigma$ is the concentration parameter that affects the *a priori* number of ones per column. When $\delta = 1$ and $\sigma = 0$, we recover the standard IBP model.

Using the usual culinary metaphor of customers entering an Indian buffet restaurant and sequentially choosing dishes from an infinite buffet, the three-parameter IBP generalizes as follows:

- Customer 1 tries Poisson(α) number of dishes.
- Customer $c + 1$ tries:
  - each dish with probability $\frac{m_k - \sigma}{c + \delta}$ for each dish that has previously been tried, where m_k is the number of customers who previously sampled from dish k.
  - Poisson $\left(\alpha \frac{\Gamma(1+\delta)\Gamma(c+\delta+\sigma)}{\Gamma(c+1+\delta)\Gamma(\delta+\sigma)}\right)$ new dishes.

In such process, the number of hidden features is expected to grow as $O(C^\sigma)$. By introducing parameters δ and σ, matrix $\mathbf{Z}$ can have a more flexible sparsity structure, regardless of the sparsity degree which is controlled by α. Compared to the Restricted IBP prior (described in the following), the 3-parameter IBP gives more flexibility regarding the feature weights, but has the disadvantage that the number of ones per-row is still a priori Poisson distributed, which might not be desirable in all situations, particularly in our analysis of international trade.

[1] Eq. 7 and 8 employ a common slight misuse of notation by ignoring the sticks position of the beta and Bernoulli processes.

B.3 Restricted Indian Buffet Process

The Restricted IBP is a recently developed model that allows an arbitrary prior distribution to be placed over the number of active features underlying each observation [3]. A natural way to build such process is to replace the underlying Bernoulli processes in the IBP by *restricted* Bernoulli processes defined as:

$$\text{R-BeP}(\mathbf{Z}_{c\bullet};\mu,g) = g\left(J_c\right)\cdot \frac{\prod_{k=1}^{\infty}\pi_k^{Z_{ck}}(1-\pi_k^{1-Z_{ck}})\mathbb{1}(\sum_K Z_{ck}=J_c)}{\sum_{Z'\in\mathcal{Z}}\prod_k \pi_k^{Z'_k}(1-\pi_k)^{(1-Z'_k)}\mathbb{1}(\sum_K Z'_k=J_c)}, \tag{9}$$

where the directing measure $\mu = \sum_k \pi_k\delta_{\theta_k}$, π_k and θ_k are the stick weight and location corresponding to each latent feature k, α is the same mass parameter present in the IBP case, and g is the a priori distribution over the number of active features per sample. The Restricted IBP can thus be formulated as:

$$\mu \sim \text{BP}(1,\alpha,H), \tag{10}$$

$$\mathbf{Z}_{c\bullet} \sim \text{R-BeP}(\mu,g). \tag{11}$$

We thus have two degrees of freedom α and g to control for sparsity degree and sparsity structure in matrix $\mathbf{Z}$. Note that columns are not exchangeable anymore, i.e., the parameter g creates correlation among the features, which has to be dealt with during inference.

C Sparse Three-parameter Restricted IBP

To decouple sparsity degree and sparsity structure in the latent matrix, we combine the advantages of both the Restricted IBP and three-parameter IBP into a single prior,

$$\mathbf{Z} \sim \text{3R-IBP}(\alpha,\delta,\sigma,g), \tag{12}$$

where the mass parameter α controls the sparsity degree of matrix $\mathbf{Z}$, δ is the concentration parameter that accounts for the degree of sharing between features, σ is the stability exponent responsible for the power-law behavior of the stick weights, and g is the a priori distribution over the number of ones per row.

Now, let $\mathbf{M}\in\mathbb{N}^{C\times P}$ be our input matrix of C samples and P dimensions. Using the three-parameter Restricted IBP prior, we build an infinite latent feature model for count data with Poisson likelihood and Gamma-distributed factors as follows

$$M_{cp} \sim \text{Poisson}\big(\mathbf{Z}_{c\bullet}\mathbf{B}_{\bullet p}\big), \tag{13}$$

$$B_{kp} \sim \text{Gamma}\left(\alpha_B,\frac{\mu_B}{\alpha_B}\right), \tag{14}$$

$$\mathbf{Z} \sim \text{3R-IBP}(\alpha,\delta,\sigma,g) \tag{15}$$

where α_B and μ_B are the shape and mean parameters of the prior Gamma distribution for each element of matrix $\mathbf{B}$. In this model, both matrices $\mathbf{Z}$ and $\mathbf{B}$ are non-negative and sparse, which makes the inferred latent variables very easy to interpret. In particular, sparsity in matrix $\mathbf{B}$ can be induced simply by choosing $\alpha_B \ll 1$.

In our particular application of international trade, we have C countries, P products and K^+ non-empty latent features to be inferred (which, in the spirit of economic complexity, we may refer to as capabilities). A given row $\mathbf{Z}_{c\bullet}$ captures which latent features are active for country c. On the other hand, matrix $\mathbf{B}$ represents the effect of each latent feature on every product. For instance, if a latent feature k is active for a certain country, all products having high values in vector $\mathbf{B}_{k\bullet}$ will be more likely to be exported by that country.

To help further in the interpretation of features, we incorporate a *bias term* F0 into the model by forcing the first latent feature to be active for all countries[2]. By doing so, we are able to capture the average export probability for each product. Such approach has already been followed in [7] and [6] to alleviate identifiability problems in the inferred solution. This model can be seen as a probabilistic extension of non-negative matrix factorization where i) the number of latent features is not fixed a priori, ii) both matrices are sparse, and iii) soft-constraints on the expected latent sparsity structure are imposed through the prior.

[2]This feature may be seen as a basic capability present in every country.

D Inference and Settings

D.1 Inference

Since exact computation of the posterior distribution for the latent variables is intractable, we resort to a Markov Chain Monte Carlo (MCMC) approach. Specifically, we use Gibbs sampling together with Metropolis-Hasting (MH). We utilize a finite-dimensional approximation for the latent measure $\boldsymbol{\pi}$ by allowing at most K features.

For each observation M_{cp}, we introduce the auxiliary variables $M^{'}_{cp,1}, \ldots, M^{'}_{cp,K}$ such that $M_{cp} = \sum_{k=1}^{K} M^{'}_{cp,k}$, and $M^{'}_{cp,k} \sim \text{Poisson}(Z_{ck}B_{kp})$ for $k = 1, \ldots, K$. Given such auxiliary variables, the model is conditionally conjugate, and a Gibbs sampler can be derived straightforwardly. The complete sampling algorithm is described in Algorithm 1.

Algorithm 1 A single iteration of the MCMC inference procedure for the S3R-IBP model.

Sample each element of matrix $\mathbf{Z}$ using inclusion probabilities [1, 3].
Sample latent measure $\boldsymbol{\pi}$ using MH steps [3].
Sample each element of $\mathbf{B}$ and $M^{'}$ from their conditional distributions.
Sample hyperparameter α according to [2].

D.2 Simulation Settings

We consider 10 different initializations for our model. For each simulation, there is a burn-in period for the MCMC inference algorithm of 10,000 iterations. After that, we average our results using 1,000 additional posterior samples. Regarding the parameters in our model, we choose $g = \text{Negative-Binomial}(r, q)$, with $r = [1, 2]$, and $q = [0.1, 0.3, 0.5]$. The results are equivalent using any of these priors. Here, we report the results for $r = 1$ and $q = 0.1$.

Additionally, we run experiments for each combination of $n = [1, 10, 20, 50]$ and $\sigma = [0, 0.25, 0.5, 0.75, 1]$. Even if the results did not vary considerably when changing those hyperparameters, setting $n > 1$ and $\sigma > 0$ allows for a higher *a priori* sparseness in the latent features and potential power-law behaviors in the stick weights respectively. All figures and tables correspond to $n = 50$, and $\sigma = 1$. The hyperparameters for the Gamma prior over α are shape and scale equal to one. Finally, α_B is set to 0.01 to induce sparsity, and μ_B is equal to 1.

E Robustness Results

We test the robustness of our results by comparing them with: i) results from Singular Value Decomposition (SVD) - a baseline factorization method; ii) results corresponding to the year 1995; and iii) results estimated by using the Harmonized System (HS) rev. 1992 classification disaggregated to six digit level. For an adequate comparison between all results, Table A presents a more complete representation than Figure 1 in the main text. For each capability k, we report the averaged number of countries $\bar{m}_k$ that have it, the top-5 products with highest weights B_{kp} and a *representative country*, which we define as the country that has the least number of active capabilities among those that possess capability k. We also report the average number of active capabilities $\bar{J}_c$ for each representative country c.

E.1 Comparison with SVD

In Table B we report the Top-5 products with sorted highest weights from the Top-15 features learned via SVD. By comparing SVD to S3R-IBP, it is evident that our model is able to give much shorter and concise descriptions, as weights decrease at a faster pace and the largest weights are considerably larger. Moreover, the products listed in each feature of SVD come from a mixture of several different production elements (e.g. F2 includes musical accessories, friction materials, vegetables, meat and even metal products), whereas the S3R-IBP list is much more homogeneous. Thus, it is easy to conclude that our approach enhances interpretability of the latent factors in terms of both conciseness and precision, when compared to a baseline matrix factorization method.

E.2 Comparison across years

Table C presents the results for the year 1995 estimated with our model. The results are very similar as the ones presented in Table A. For instance, feature F4 that was learned in 2010 corresponds to feature F3 learned in 1995, whereas feature F11 corresponds to the same products (e.g., vehicles) in both years. Additionally, features that are present in most countries (except the bias) appear in both years. Although labels might be switched, which is to be expected in these latent feature models, we infer similar capabilities for 1995 and 2010.

E.3 Comparison across datasets

Table D displays the results for 2010 estimated for the HS classification dataset ($C = 123$, $P = 4890$). Since the number of products in this dataset is much higher than for the SITC classification, our model infers a higher number of capabilities. Indeed, the HS classification offers more information about the productive structure of economies. Despite the difference in the total number of capabilities, there is a striking resemblance among the capabilities in both cases. For example, we can clearly associate capability F2 from Table A to capability F4 from D, or the vehicle feature F11, with feature F14. Interestingly, the HS classification offers more granular capabilities to describe a single capability in the SITC case, e.g., F1, F2 and F4 in Table D corresponds to F2 in Table A.

S1 Table A. Complete List of Capabilities found by the S3R-IBP model in 2010 through the SITC classification.

Id	$\bar{m}_k$	**Top-5 products with sorted highest weights (B_{kp}) associated**	**Repr. countries ($\bar{J}_c$)**
F0	126	Non-Coniferous Worked Wood (0.40), Bran and Other Cereals Residues (0.39), Miscellaneous Non-Iron Waste (0.38), Unwrought Lead (0.38), Bones, Ivory and Horns (0.37)	-
F1	38.67	Vegetables (0.60), Fruit or Vegetable Juices (0.54), Miscellaneous Fruit (0.50), Frozen Vegetables (0.48), Apples (0.47)	Peru (2.00)
F2	46.11	Synthetic Knitted Undergarments (0.76), Miscellaneous Feminine Outerwear (0.74), Miscellaneous Knitted Outerwear (0.73), Men's Shirts (0.70), Blouses (0.67)	Sri Lanka (2.00)
F3	18.27	Miscellaneous Animal Oils (0.78), Bovine and Equine Entrails (0.72), Bovine meat (0.68), Preserved Milk (0.63), Equine (0.62)	Paraguay (2.00)
F4	21.39	Synthetic Woven Fabrics (0.74), Non-retail Synthetic Yarn (0.60), Woven Fabric of less than 85% Discontinuous Synthetic Fibres (0.60), Woven Fabrics of More Than 85% Discontinuous Synthetic Fiber (0.58), Yarn of Less Than 85% Synthetic Fibers (0.53)	United Arab Emirates (2.82)
F5	16.53	Miscellaneous Electrical Machinery (0.76), Vehicles Stereos (0.72), Miscellaneous Data Processing Equipment (0.64), Video and Sound Recorders (0.57), Calculating Machines (0.55)	Malaysia (3.00)
F6	45.93	Baked Goods (0.67), Metal Containers (0.62), Miscellaneous Edibles (0.59), Miscellaneous Articles of Paper (0.59), Miscellaneous Organic Surfactants (0.58)	Costa Rica (2.06)
F7	21.95	Measuring Controlling Instruments (0.61), Mathematical Calculation Instruments (0.59), Miscellaneous Electrical Instruments (0.57), Miscellaneous Heating and Cooling Equipment (0.51), Parts of Office Machines (0.49)	Malaysia (3.00)
F8	33.23	Miscellaneous Articles of Iron (0.65), Carpentry Wood (0.61), Miscellaneous Manufactured Wood Articles (0.60), Sawn Wood Less Than 5mm Thick (0.56), Electric Current (0.51)	Russia (2.93)
F9	32.12	Miscellaneous Rotating Electric Plant Parts (0.66), Control Instruments of Gas or Liquid (0.58), Valves (0.57), Miscellaneous Rubber (0.56), Miscellaneous Articles of Plastic (0.55)	Philippines (4.01)
F10	33.00	Improved Wood (0.71), Mineral Wool (0.62), Central Heating Equipment (0.62), Aluminium Structures (0.62), Harvesting Machines (0.60)	Belarus (4.20)
F11	31.14	Vehicles Parts and Accessories (0.59), Cars (0.58), Iron Wire (0.53), Trucks and Vans (0.53), Air Pumps and Compressors (0.50)	Belarus (4.20)
F12	11.04	Synthetic Rubber (0.87), Acrylic Polymers (0.85), Silicones (0.76), Miscellaneous Polymerization Products (0.71), Tinned Sheets (0.65)	North Korea (3.99)
F13	18.67	Aldehyde, Ketone and Quinone-Function Compounds (0.68), Glycosides and Vaccines (0.67), Medicaments (0.65), Inorganic Esters (0.64), Cyclic Alcohols (0.62)	Ireland (4.34)
F14	14.87	Parts of Metalworking Machine Tools (0.74), Interchangeable Tool Parts (0.72), Polishing Stones (0.69), Tool Holders (0.66), Miscellaneous Metalworking Machine-Tools (0.54)	Israel (5.97)
F15	23.29	Miscellaneous Pumps (0.51), Ash and Residues (0.45), Chemical Wood Pulp of sulphite (0.44), Rolls of Paper (0.43), Worked Nickel (0.43)	Russia (2.93)

From left to right, $\bar{m}_k$ is the averaged number of countries having latent feature k active, we list the top-5 products with highest weights B_{kp}; a *representative country* is the country that has the least number of capabilities among those possessing feature k. $\bar{J}_c$ is the averaged number of active features for each representative country c.

S1 Table B. Top-15 latent features inferred using the Singular Value Decomposition through the SITC classification.

Id	**Top-5 products with sorted highest weights (B_{kp}) associated**
F1	Miscellaneous Non-Ferrous Ores (0.40), Petroleum Gases (0.40), Miscellaneous Textile Articles (0.37), Zinc Ore (0.32), Miscellaneous Bituminous Mixtures (0.31)
F2	Sound Recording Media (0.38), Asbestos Products (0.38), Potatoes (0.37), Silver (0.35), Pig Meat (0.32)
F3	Thin Iron Sheets (0.42), Miscellaneous Food-Processing Machinery (0.41), Baked Goods (0.41), Miscellaneous Animal Entrails (0.34), Basketwork (0.34)
F4	Perfumery and Cosmetics (0.45), Miscellaneous Gas Turbines (0.38), Cut Paper (0.35), Miscellaneous Cereal Grains (0.33), Herbicides (0.32)
F5	Bovine (0.49), Miscellaneous Refrigeration Equipment (0.43), Radioactive Chemicals (0.41), Blocks of Iron and Steel (0.41), Rape Seeds (0.40)
F6	Wheat Flour (0.34), Iron and Steel Forging (0.29), Printing Ink (0.29), Waste Paper (0.28), Aluminum (0.26)
F7	Miscellaneous Oil Seeds and Fruits (0.47), Bones, Ivory and Horns (0.44), Temporarily Preserved Fruit (0.43), Cotton Seed Oil (0.42), Inorganic Bases (0.39)
F8	Prepared Explosives (0.48), Confectionary Sugar (0.39), Cigarretes (0.38), Coke (0.37), Miscellaneous Hides and Skins (0.34)
F9	Fish, preserved (0.44), Fresh Fish (0.43), Miscellaneous Animal Origin Materials (0.40), Oranges (0.37), Sheep and Goat Meat (0.37)
F10	Wood and Animal Hair Waste (0.46), Miscellaneous Carpets (0.42), Wool Carpets (0.41), Wool Yarn (0.40), Degreased Sheep Wool (0.38)
F11	Tin (0.41), Vehicles Stereos (0.40), Copper (0.36), Miscellaneous Articles of Paper (0.36), Petroleum Gases (0.36)
F12	Gypsum and Other Calcareous Stone (0.42), Sausage (0.34), Special Products of Textile (0.32), Movie Cameras and Equipment (0.30), Iron Shapes (0.29)
F13	Cigarretes (0.50), Worked Tin and Alloys (0.43), Aluminum (0.38), Bicycles (0.38), Raw Sheep Skin without Wool (0.38)
F14	Precious Metal Ores (0.50), Gold (0.48), Diamonds (0.47), Unmounted Precious Stones (0.43), Electrical Transformers (0.38)
F15	Sulphur (0.40), Fuel Wood and Charcoal (0.34), Miscellaneous Unmilled Cereals (0.33), Household Refrigeration (0.33), Decorative Wood (0.33)

S1 Table C. Complete List of Capabilities found by the S3R-IBP model in 1995 through the SITC classification.

Id	$\bar{m}_k$	**Top-5 products with sorted highest weights (B_{kp}) associated**	**Repr. countries ($\bar{J}_c$)**
F0	125	Miscellaneous Non-Iron Waste (0.45), Men's Shirts (0.39), Miscellaneous Live Animals (0.39), Lubricating Petroleum Oils (0.38), Raw Goat Skins (0.37)	-
F1	45.46	Fruit Jams (0.51), Baked Goods (0.49), Confectionary Sugar (0.49), Miscellaneous Metal Articles (0.47), Miscellaneous Beverages (0.46)	Jordan (2)
F2	51.18	Womens Knitted Outerwear (0.81), Womens Coats (0.8), Skirts (0.78), Mens Jackets (0.77), Miscellaneous Feminine Outerwear (0.76)	Bangladesh (2)
F3	15.22	Woven Fabric of less than 85% Discontinuous Synthetic Fibres (0.75), Synthetic Woven Fabrics (0.68), Embroidery (0.63), Fabrics of more than 85% discontinuous regenerated fibres (0.63), Miscellaneous Woven Fabrics (0.63)	Pakistan (3)
F4	13.86	Miscellaneous Electrical Machinery (0.81), Computer Peripherals (0.79), Computer Parts and Accessories (0.79), Electrical Resistors (0.68), Diodes, Transistors and Photocells (0.67)	Singapore (3)
F5	10.04	Cameras (0.93), Calculating Machines (0.83), Video and Sound Recorders (0.81), Vehicles Stereos (0.8), Recorded Audio Players (0.75)	Singapore (3)
F6	19.84	Miscellaneous Articles of Plastic (0.62), Mirrors (0.61), Plastic Lamps (0.59), Miscellaneous Non-Electrical Machinery Parts (0.53), Locksmith Hardware (0.5)	Hong Kong (6)
F7	43.7	Fiberboard (0.69), Miscellaneous Furniture (0.48), Chemical Wood Pulp of sulphite (0.46), Tissue Paper (0.46), Wood Boxes (0.45)	Zimbabwe (3.02)
F8	11.1	Organic Chemical Products (0.65), Phenols(0.58), Gas, Liquid and Electric Meters (0.57), Chemical Products (0.53), Fungicides (0.5)	Israel (6.98)
F9	40.04	Iron Coils (0.59), Thin Iron Sheets (0.54), Thick Iron Sheets (0.52), Metal Cables (0.51), Iron Wire (0.47)	Jordan (3)
F10	15.04	Valves (0.89), Industrial Furnaces and Ovens (0.7), Centrifugal Pumps (0.68), Miscellaneous Refrigeration Equipment (0.64), Pulley System Parts (0.64)	Bosnia and Herzegovina (4.02)
F11	15.14	Vehicles Parts and Accessories (0.71), Motor Vehicles Piston Engines (0.66), Miscellaneous Refractory Goods (0.63), Miscellaneous Rubber (0.58), Piston Engine Parts (0.53)	Mexico (6.06)
F12	22	Miscellaneous Mineral Materials (0.78), Mineral Wool (0.73), Varnishes and Lacquers (0.64), Glass Fiber Fabrics (0.64), Miscellaneous Printed Matter	Norway (4)
F13	12.06	Amine-Function Compounds (0.88), Inorganic Esters (0.73), Oxygen-Function Amino-Compounds (0.66), Organo-Sulphur Compounds (0.65), Glues (0.63)	Israel (6.98)
F14	23.04	Iron Tubes (0.83), Miscellaneous Machinery (0.64), Fasteners (0.60), Miscellaneous Parts of Lifting Machinery (0.59), Miscellaneous Iron Tubes and Pipes (0.55)	Georgia (2)
F15	14.74	Interchangeable Tool Parts (0.74), Miscellaneous Pump Parts (0.67), Miscellaneous Liquid Pump Parts (0.66), Miscellaneous Printing Machines (0.64), Miscellaneous Agricultural Machinery (0.62)	Israel (6.98)
F16	23.26	Iron Ore Agglomerates (0.56), Ferro-alloys (0.53), Prepared Explosives (0.53), Iron Ore (0.49), Inorganic Bases (0.47)	Venezuela (2.12)

From left to right, $\bar{m}_k$ is the averaged number of countries having latent feature k active, we list the top-5 products with highest weights B_{kd}; a *representative country* is the country that has the least number of capabilities among those possessing feature k. $\bar{J}_c$ is the averaged number of active features for each representative country n.

S1 Table D. Complete List of Capabilities found by the S3R-IBP model in 2010 through the HS classification.

Id	$\bar{m}_k$	**Top-5 products with sorted highest weights (B_{kp}) associated**	**Repr. countries ($\bar{J}_c$)**
F0	123	Waste or scrap, aluminium (0.39), Wheat bran, sharps, other residues (0.39), Lumber, non-coniferous nes (0.37), Oils petroleum, bituminous, distillates, except crude (0.37), Lead unwrought containing mostly antimony (0.37)	-
F1	44	Mens, boys trousers & shorts, material nes, not knit (0.72), Womens, girls blouses & shirts, material nes, not knit (0.72), Mens, boys jackets & blazers, material nes, not knit (0.72), Womens, girls skirts, of material nes, not knit (0.71), Womens, girls blouses & shirts, of material nes, knit (0.69)	Ethiopia (2)
F2	40.26	Womens, girls suits, of wool or hair, not knit (0.64), Womens, girls dresses, of wool or hair, knit (0.56), Womens, girls overcoats, etc, of wool or hair, knit (0.55), Mens, boys suits, of materials nes, knit (0.52), Womens, girls garments nes, of wool or hair, not knit (0.51)	Australia (3)
F3	38.77	Ammonium nitrate limestone etc mixes, pack >10 kg (0.54), Plastic builders ware nes (0.53), Scarifiers, cultivators, weeders and hoes (0.52), Waste or scrap, of stainless steel (0.51), Furniture parts nes (0.51)	Albania (4)
F4	37.98	Womens, girls blouses & shirts, of cotton, knit (0.8), Mens, boys shirts, of cotton, knit (0.75), Pullovers, cardigans etc of cotton, knit (0.75), Womens, girls trousers & shorts, of cotton, knit (0.73), Mens, boys shirts, of manmade fibres, knit (0.71)	Ethiopia (2)
F5	34	Parts for electric motors and generators (0.62), Electric heating resistors (0.59), Parts of cycle & vehicle light, signal, etc equipment (0.59), Parts of electrical transformers and inductors (0.59), Lock parts, etc, of base metal (0.56)	Moldova (5)
F6	31.31	Polymer based paints & varnishes nes, aqueous medium (0.46), Synthetic organic products used as luminophores (0.43), Chain and parts thereof of copper (0.41), Insulated winding wire, nes (0.38), Boxes, moulding, for metal foundry (0.36)	Albania (4)
F7	30	Bars, rods and other profiles, aluminium alloyed (0.64), Plastic doors and windows and frames thereof (0.64), Wooden cases, boxes, crates, drums and containers (0.63), Angles/shapes/sections, iron or non-alloy steel, nes (0.61), Sheet etc, cellular of polymers of styrene (0.60)	Moldova (5)
F8	28	Springs, iron or steel, except helical/leaf (0.59), Foil, aluminium, not backed, rolled but nfw, <0.2mm (0.52), Chain, iron or steel, nes (0.50), Railway passenger and special purpose coaches (0.45), Parts, accessories nes, metal cutting machine tools (0.43)	Norway (3.43)
F9	26.88	Trailer/non-mechanically propelled vehicle parts nes (0.65), Milk and cream not concentrated nor sweetened <6% fa (0.65), Poultry, domestic, whole, fresh or chilled (0.65), Sausages, similar products of meat, meat offal & bloo (0.64), Milk not concentrated nor sweetened 1-6% fat (0.63),	Ireland (4.34)
F10	25	Drinking glasses, except lead crystal or glass ceramic (0.61), Refractory bricks etc >50% alumina or silica (0.55), Wheels including parts/accessories for motor vehicles (0.54), Woven fabric polyester + wool or hair, nes (0.52), Glass mirrors, unframed (0.52)	Australia (3)
F11	24	Textile products and articles for technical uses, nes (0.59), Parts, laboratory/industrial heating/cooling machinery (0.57), Medical, surgical or laboratory sterilizers (0.54), Electrical relays for 60 - 1,000 volts (0.54), Electrodes etc of base metal or metal carbide, nes (0.51)	Canada (8)
F12	24	Parts agricultural, forestry, bee-keeping machines nes (0.78), Parts of agricultural machinery (0.62), Parts for soil preparation or cultivation machinery (0.60), Rye (0.59), Hydraulic power engines/motors, linear acting (0.57)	Norway (3.43)

S1 Table D Continued.

Id	$\bar{m_k}$	Top-5 products with sorted highest weights (B_{kp}) associated	Repr. countries ($\bar{J_c}$)
F13	23	Cranes or derricks nes (0.66), Bars, rods & profiles of copper-zinc base alloys (0.62), Polishes, creams etc. for maintenance of woodwork (0.54), Num controlled machine tools to bend, fold, etc, meta (0.54), Acrylic & vinyl polymer based paint, varnish, in water (0.51)	South Africa (7.39)
F14	22	Motor vehicle parts nes (0.77), Parts and accessories of bodies nes for motor vehicle (0.68), Rubber tube, pipe, hose textile-reinforced no fitting (0.67), Medium Diesel Engine Cars (0.65), Motor vehicle mountings, fittings, of base metal, nes (0.64)	Canada (8)
F15	22	Embroidery of natural textile fibres except cotton (0.67), Woven fabric>85% synth nes+cotton, >170g/m2 unbl/blch (0.64), Woven fabric>85% synth nes + cotton,<170g/m2 yarn dye (0.63), Unglazed ceramic mosaic tiles etc, <7cm wide (0.60), Carpets of yarn nes, woven pile, not made up, nes (0.55)	Albania (4)
F16	17.91	Grinding/polishing machines for stone, ceramics, glass (0.55), Dividing heads/attachments nes for machine tools (0.53),Tools for milling (0.53), Industrial electric resistance heated furnaces & oven (0.52), Blades for kitchen appliances & food industry machine (0.51)	Estonia (11)
F17	17	Sheet/film not cellular/reinf amino-resins (0.57), Anti-oxidisers and stabilizers for rubber or plastics (0.51), Nitrile-function compounds, nes (0.42), Esters of inorganic acids, nes, their salts, derivs (0.41), Parts and accessories for drawing, etc instruments (0.41)	Canada (8)
F18	16.77	Parts of milking machines and dairy machinery (0.67), Eels, fresh or chilled, whole (0.63), Industrial machinery nes for food, drink preparation (0.61), Cod, fresh or chilled, whole (0.59), Chemical industry products, preparations, mixtures (0.53)	Moldova (5)
F19	16	Gasket sets, other joints of similar composition (0.65), Lubricating preparations, zero petroleum content, nes (0.60), Spectrometers, spectrophotometers, etc using light (0.57), Staple fibres of nylon, polyamides, not carded, combe (0.52), Organo-sulphur compounds, nes (0.51)	Singapore (9)
F20	15.4	Synthetic organic tanning substances (0.69), Citrus fruits, otherwise prepared or preserved (0.54), Other finishing agents of a kind used in the leather or like industries (0.53), Inorganic tanning and pre-tanning preparations (0.53), Citrus juice nes (one fruit) not fermented or spirite (0.5)	Australia (3)
F21	15.27	Heterocyclic compound,oxygen hetero-atom(s) only,nes (0.73), Heterocyclic compounds with unfused pyridine ring, nes (0.71), Lactones, other than coumarins (0.59), Printing ink, other than black (0.59), Heterocyclic compds with an unfused triazine ring nes (0.58)	Ireland (4.34)
F22	15	Tungsten unwrought, bars/rods simply sintered, scrap (0.63), Mica plates, sheets and strips (0.6),Parts and accessories for flashlights and flashbulbs (0.55), Circular saw blades, working part other than steel (0.52), Base metals clad with silver, semi-manufactured (0.49)	Estonia (11)
F23	14	Horses, live except pure-bred breeding (0.82), Bovine cuts boneless, fresh or chilled (0.71), Cheese, grated or powdered, of all kinds (0.70), Bovine livers, frozen (0.7), Seed, rye grass, for sowing (0.70)	Australia (3)
F24	14	Olives, prepared or preserved, not frozen/vinegar (0.75), Olive oil, fractions, blends, not chemically modified (0.70), Olive oil, fractions, refined, not chemically modifie (0.70), Tomatoes nes, prepared or preserved, not in vinegar (0.70), Olive oil, virgin(0.68)	Belarus (6)

S1 Table D Continued.

Id	$\bar{m}_k$	Top-5 products with sorted highest weights (B_{kp}) associated	Repr. countries ($\bar{J}_c$)
F25	13	Woven cotton nes, <85% +manmade fibre, <200g/m2 dyed (0.8), Woven fabric synthetic filament <85% +cotton, dyed ne (0.79), Plain weave cotton, >85% 100-200g/m2, dyed (0.75), Woven cotton nes, >85% <200g/m2, printed (0.71), Woven cotton nes, >85% >200g/m2, dyed, nes (0.69)	Moldova (5)
F26	13	Belts and bandoliers of leather or composition leather (0.84), Woven fabric synthetic staple fibre with manmade, nes (0.71), Sheep or lamb skin leather, nes (0.68), Twill cotton except denim, >85% >200g/m2, yarn dyed (0.63), Woven fabric <85% artificial staple+manmade fibre dye (0.59)	Belarus (6)
F27	12	Cotton yarn <85% single uncombed >714dtex, not retail (0.85), Cotton yarn <85% multiple combed <125 dtex, not retai (0.75), Cotton yarn <85% single combed >714 dtex, not retail (0.71), Cotton yarn <85% single combed 192-125 dtex,not retai (0.70), Cotton yarn <85% single combed <125 dtex, not retail (0.70)	Canada (8)
F28	11.04	Adipic acid, its salts & esters (0.64), Diols except ethylene and propylene glycol (0.62), Coated rods/cored wire for flame solder/braze/weld (0.61), Film in rolls, width 105-610 mm nes (0.60), Acrylic acid esters (0.59)	Belarus (6)
F29	10	Parts and accessories of recorders except cartridges (1.02), Cast, drawn or float glass sheet, edge worked or bent (0.84), Soya sauce (0.84), Electri capacitors, fixed, ceramic, single layer (0.83), Permanent magnets & articles intended as magnets, nes (0.79)	Moldova (5)
F30	9	1-chloro-2,3-epoxypropane(epichlorohy-drin) (0.76), Dichloromethane (methylene chloride) (0.74), Halides & halide oxides of non-metals (not chlorides) (0.64), Chloroform (trichloromethane) (0.64), Cresols, salts (0.61)	Albania (4)
F31	6	Bituminous coal, not agglomerated (0.81), Balls, iron/steel, forged/stamped for grinding mills (0.77), Iron ore, concentrate, not iron pyrites, agglomerated (0.61), Residual lyes from the manufacture of wood pulp (0.60), Palladium in semi-manufactured forms (0.56)	Australia (3)
F32	6	Vinyl polymers, halogenated olefins, primary form, ne (0.89), Polyvinyl alcohols in primary form (0.64), Methyloxirane (propylene oxide)(0.63), Ultra-violet or infra-red lamps, arc lamps (0.57), Tin alloys unwrought (0.56)	Singapore (9)
F33	6	Sound reproducing apparatus, non-recording (0.85), Radio receivers, portable, with sound reproduce/recor (0.80), Erasers (vulcanised rubber) (0.76), Multiple loudspeakers, mounted in single enclosure (0.76), Telephone sets (0.70)	Canada (8)
F34	5	Paper, filter, cut to size or shape (0.77), Carbon tetrachloride (0.61), Cellulose acetates, non-plasticised, in primary forms (0.54), Polyvinyl chloride nes, not plasticised, primary form (0.50), Spacecraft, satellites and spacecraft launch vehicles (0.49)	United Kingdom (19)
F35	4	Articles for Christmas festivities (1.05), Parts nes, for dolls representing only human beings (0.96), Dolls representing only human beings (0.91), Lighting sets of a kind used for Christmas trees (0.89), Stuffed toys - animals or non-human creatures (0.88),	Moldova (5)
F36	3	Antimony, articles thereof, waste or scrap (0.91), Electric toasters, domestic (0.91), Manganese, articles thereof, waste or scrap (0.79), Rare-earth metals, scandium and yttrium (0.68), Fabric impregnated, coated, covered with polyurethane (0.66)	Singapore (9)
F37	3	Molybdenum concentrates, roasted (0.73), Molybdenum ores and concentrates except roasted (0.70), Cameras for 35 mm roll film except single lens reflex (0.63), Lettuce, fresh or chilled except cabbage lettuce (0.61), Propionic acid, its salts & esters (0.55),	Canada (8)

S1 Table D Continued.

Id	$\bar{m_k}$	**Top-5 products with sorted highest weights (B_{kp}) associated**	**Repr. countries ($\bar{J}_c$)**
F38	2	Chlorobenzene, o-dichlorobenzene and p-dichlorobenzen (0.59), Cresols, salts (0.53), Aminohydroxynaphthalenesulphonic acids and salts (0.5), Diphenylamine, derivatives, salts thereof (0.49), Zinc sulphide (0.48)	India (18)
F39	1	Parts of cathode-ray tubes (0.51), Moulds for rubber or plastic, nes (0.50), Bolts/screws nes, with/without nut/washer, iron/steel (0.46), Plates, sheet, strip and foil, nickel, not alloyed (0.45), Cotton yarn $<85\%$ single uncombed >714dtex, not retail (0.45),	Moldova (5)
F40	1	Parts and accessories for photo-copying apparatus (0.52), Wagon handling equipment (0.47), Thread rolling machines for working metal, etc (0.46), Adipic acid, its salts & esters (0.46), Xylenols, salts (0.44)	Canada (8)
F41	1	Powders, alloy steel (0.46), Injection-moulding machines for rubber or plastic (0.43), Chem wood pulp, sulphite, non-coniferous, unbleached (0.43), Apparatus for electro-plating, electrolysis, etc (0.42), Spinning spindles, spindle flyers, spinning rings (0.41)	Canada (8)
F42	1	Seed, rye grass, for sowing (0.44), Pneumatic hand tool parts (0.41), Bed linen, of material nes (0.41), Bird skins and feathers, articles therefrom (0.39), Cartridges for rivet etc tools, humane killers, etc(0.38)	South Africa (7.39)

From left to right, $\bar{m_k}$ is the averaged number of countries having latent feature k active, we list the top-5 products with highest weights B_{kd}; a *representative country* is the country that has the least number of capabilities among those possessing feature k. $\bar{J}_c$ is the averaged number of active features for each representative country n.

Bibliography

[1] Nibia Aires. "Algorithms to find exact inclusion probabilities for conditional Poisson sampling and Pareto sampling designs". In: *Methodology and Computing in Applied Probability* 1.4 (1999), pp. 457–469.

[2] Cedric Archambeau, Balaji Lakshminarayanan, and Guillaume Bouchard. "Latent IBP compound Dirichlet allocation". In: *IEEE transactions on pattern analysis and machine intelligence* 37.2 (2015), pp. 321–333.

[3] Finale Doshi-Velez and Sinead A. Williamson. "Restricted Indian Buffet Processes". In: *arXiv preprint arXiv:1508.06303* (2015).

[4] Thomas L. Griffiths and Zoubin Ghahramani. "The Indian Buffet Process: An Introduction and Review." In: *Journal of Machine Learning Research* 12 (2011), pp. 1185–1224.

[5] César A Hidalgo et al. "The product space conditions the development of nations". In: *Science* 317.5837 (2007), pp. 482–487.

[6] Melanie F. Pradier, Francisco J. R. Ruiz, and Fernando Perez-Cruz. "Prior Design for Dependent Dirichlet Processes: An Application to Marathon Modeling". In: *PLOS ONE* 11.1 (Jan. 2016), e0147402. ISSN: 1932-6203. DOI: 10.1371/journal.pone.0147402.

[7] Francisco J. R. Ruiz et al. "Bayesian Nonparametric Comorbidity Analysis of Psychiatric Disorders". In: *J. Mach. Learn. Res.* 15.1 (Jan. 2014), pp. 1215–1247. ISSN: 1532-4435.

[8] Yee W. Teh and Dilan Gorur. "Indian Buffet Processes with Power-law Behavior". In: *Advances in Neural Information Processing Systems 22*. Ed. by Y. Bengio et al. Curran Associates, Inc., 2009, pp. 1838–1846.

[9] Romain Thibaux and Michael I. Jordan. "Hierarchical beta processes and the Indian buffet process". In: *International Conference on Artificial Intelligence and Statistics*. 2007, pp. 564–571.
